# Structure of Lacticaseicin 30 and Its Engineered Variants Revealed an Interplay between the N-Terminal and C-Terminal Regions in the Activity against Gram-Negative Bacteria

**DOI:** 10.3390/pharmaceutics14091921

**Published:** 2022-09-12

**Authors:** Désiré Madi-Moussa, Barbara Deracinois, Radja Teiar, Yanyan Li, Marius Mihasan, Christophe Flahaut, Sylvie Rebuffat, Françoise Coucheney, Djamel Drider

**Affiliations:** 1 UMR Transfrontalière BioEcoAgro1158, Univ. Lille, INRAE, Univ. Liège, UPJV, JUNIA, Univ. Artois, Univ. Littoral Côte d’Opale, ICV—Institut Charles Viollette, 59000 Lille, France; 2 Laboratory Molecules of Communication and Adaptation of Microorganisms (MCAM, UMR 7245 CNRS-MNHN), National Museum of Natural History (MNHN), CNRS, CP 54, 57 Rue Cuvier, 75005 Paris, France; 3 Biochemistry and Molecular Biology Laboratory, Faculty of Biology, Alexandru Ioan Cuza University of Iasi, Carol I Blvd., No. 20A, 700506 Iasi, Romania

**Keywords:** *Escherichia coli*, antimicrobial activity, structure-activity relationship, helical conformation

## Abstract

Lacticaseicin 30 is one of the five bacteriocins produced by the Gram-positive *Lacticaseibacillus paracasei* CNCM I-5369. This 111 amino acid bacteriocin is noteworthy for being active against Gram-negative bacilli including *Escherichia coli* strains resistant to colistin. Prediction of the lacticaseicin 30 structure using the Alphafold2 pipeline revealed a largely helical structure including five helix segments, which was confirmed by circular dichroism. To identify the structural requirements of the lacticaseicin 30 activity directed against Gram-negative bacilli, a series of variants, either shortened or containing point mutations, was heterologously produced in *Escherichia coli* and assayed for their antibacterial activity against a panel of target strains including Gram-negative bacteria and the Gram-positive *Listeria innocua*. Lacticaseicin 30 variants comprising either the N-terminal region (amino acids 1 to 39) or the central and C-terminal regions (amino acids 40 to 111) were prepared. Furthermore, mutations were introduced by site-directed mutagenesis to obtain ten bacteriocin variants E6G, T7P, E32G, T33P, T52P, D57G, A74P, Y78S, Y93S and A97P. Compared to lacticaseicin 30, the anti-Gram-negative activity of the N-terminal peptide and variants E32G, T33P and D57G remained almost unchanged, while that of the C-terminal peptide and variants E6G, T7P, T52P, A74P, Y78S, Y93S and A97P was significantly altered. Finally, the N-terminal region was further shortened to keep only the first 20 amino acid part that was predicted to include the first helix. The anti-Gram-negative activity of this truncated peptide was completely abolished. Overall, this study shows that activity of lacticaseicin 30, one of the rare Gram-positive bacteriocins inhibiting Gram-negative bacteria, requires at least two helices in the N-terminal region and that the C-terminal region carries amino acids playing a role in modulation of the activity. Taken together, these data will help to design forthcoming variants of lacticaseicin 30 as promising therapeutic agents to treat infections caused by Gram-negative bacilli.

## 1. Introduction

The antibiotic crisis is now well acknowledged worldwide as a serious health problem. Antibiotics which enabled saving millions of lives in the world since their discovery are now facing a rapid decrease in efficiency due to the bacterial resistance crisis. The reasons usually reported to explain such a situation include the overuse and misuse of conventional antibiotics, and their inappropriate prescription. Moreover, possibilities to refill the antibiotic pipeline will be very limited in the near future due to reduced economic incentives expected by the major pharmaceutical companies [1,2]. In 2014, antimicrobial resistance (AMR) was estimated to cause 10 million deaths per year by 2050 [3]. To face this overwhelming situation, numerous alternatives to antibiotics have been explored, among which bacteriophage therapy [4,5], predatory bacteria [6], competitive exclusion of pathogens [7] and bacteriocins [8,9,10] are included. These approaches offer clear advantages, such as their specificity and low detrimental impact on beneficial microbial communities, unlike antibiotics which generally have collateral damage on commensal bacteria [11]. 

Bacteriocins are ribosomally synthesized antimicrobial peptides (AMPs) produced by Gram-positive and Gram-negative bacteria as well as Archaea [12,13]. They exhibit extensive variations in their molecular masses, inhibitory spectra, modes of action, and mechanisms of biosynthesis, export and self-protection of the producing strains [14]. These AMPs are considered to be significant actors of microbial competitions because of their role in colonizing niches, killing competing strains and their use of cross-talk or quorum-sensing networks within bacterial communities [15,16,17]. Bacteriocins from Gram-positive bacteria are predominantly produced by lactic acid bacteria (LAB). These bacteriocins, referred to here as LAB-bacteriocins, are safe for cells from the Eukarya domain [18,19]. They show most often a narrow spectra of activity, acting therefore selectively on members of species identical or closely related to the producer, and in rarer cases they exhibit broad spectra, thus targeting other species [20,21]. During the last few years, several classifications of LAB-bacteriocins have been proposed [17,22,23]. Among those, the classification proposed by Alvarez-Sieiro et al. [22], dividing LAB-bacteriocins into three main classes, is largely used. According to this classification, the class I of bacteriocins includes peptides that have undergone extensive post-translational modifications during their biosynthesis, resulting in the introduction of rare amino acids, such as lanthionines that are present in lanthipeptides. Class II includes unmodified bacteriocins having molecular masses below 10 kDa, while class III contains thermo-labile unmodified bacteriocins of more than 10 kDa with a bacteriolytic or non-lytic mechanism of action [23]. Depending on their structural and functional characteristics, many LAB-bacteriocins act on the cytoplasmic membrane of target bacteria by forming pores, leading to the leakage of ions and small essential molecules, or by degrading the cell walls. With continuing research, LAB-bacteriocins have been allocated with further functions such as antiviral activity or inhibition of proliferation of unscheduled and unregulated tumor cell lines [17,24]. Gram-negative bacteria are generally resistant to LAB-bacteriocins due to their outer membrane, which confers upon them supplementary protection against antimicrobial agents. Nonetheless, a limited number of LAB-bacteriocins possessing activity against Gram-negative bacteria, including *Escherichia coli,* have been reported in the literature [25,26,27]. However, if the mode of action of LAB-bacteriocins against Gram-positive bacteria is globally well documented [8,28], while their action against Gram-negative bacteria remains to be understood.

Here, we focused on *Lacticaseibacillus paracasei* CNCM I-5369, a strain isolated from an Algerian dairy product recently shown to produce five class II bacteriocins endowed with activity at pH 5 against Gram-negative bacteria including *E. coli* strains resistant to colistin [29]. Moreover, we have heterologously produced each of these bacteriocins, which are encoded by *orf010*, *orf012*, *orf023*, *orf030* and *orf038*. The bacteriocin encoded by *orf030*, here referred to as lacticaseicin 30, was obtained in large quantities in the soluble fraction, contrarily to the other produced peptides [30], and was shown to exhibit potent activity against *E. coli*.

In this study, the structure-activity relationship of lacticaseicin 30 was investigated. For this purpose, the predicted secondary structure of this bacteriocin, which includes five helices distributed over the 111 amino acid sequence, was used to design a series of variants, exhibiting either truncated sequences including one helix, two helices or three helices, or specific point mutations. All lacticaseicin 30 variants were assessed for their anti-Gram-negative activity.

## 2. Materials and Methods 

### 2.1. Bacterial Strains, Plasmids and Culture Conditions

Bacterial strains and plasmids used in this work are given in the supplementary materials (Table S1). *E. coli* strains were grown in Luria–Bertani (LB) broth [31] at 37 °C, with shaking at 160 rpm and, when necessary, ampicillin at 100 μg/mL (Sigma Aldrich, St Louis, MO, USA) was added to the medium. Strains used as target organisms to assess antibacterial activity were cultivated, without shaking, in brain heart infusion (BHI, Sigma Aldrich, Saint Louis, MO, USA) medium at 37 °C for 12–18 h before use.

### 2.2. Construction of Lacticaseicin 30 Variant Peptides Carrying N-Terminal Part (N-Ter Lacticaseicin 30), or the Central and C-Terminal Parts (C-Ter Lacticaseicin 30) and Their Expression in E. coli Cells

All oligonucleotides used in this work are listed in the supplementary materials (Table S2). The molecular cloning and other standard techniques were used thereof to perform genetic construction of lacticaseicin 30 and variants. N-ter-lacticaseicin 30, C-ter lacticaseicin 30 and N-ter-H1 lacticaseicin 30 plasmids were described by Sambrook et al. [32]. The *orf030*, *orf030-nter*, *orf030-cter* and *orf030-nter-h1* were amplified by PCR using pT7-6his-030 plasmid as template and F-*Bam*HI-030 and R-030-*Hind*III (for *orf030*), F-*Bam*HI-030 and R-Nter_030-*Hind*III (for *orf030-nter*), F-*Bam*HI-Cter_030 and R-030-*Hind*III (for *orf030-cter*) and F-*Bam*HI-030 and R-Nter-H1_030-*Hind*III (for *orf030-nter-h1*) primers. Then, each PCR product was cloned between the *Bam*HI and *Hind*III sites of the pET-32b(+) plasmid. 

Phusion High-Fidelity DNA Polymerase, restriction endonucleases and T4 ligase were obtained from ThermoFisher Scientific (Thermo Scientific, Waltham, MA, USA) and used in accordance with the manufacturer’s instructions. Plasmids and PCR products were purified using NucleoSpin kits (Macherey-Nagel, Düren, Germany) and the final plasmid constructions were verified by PCR and sequencing (Eurofins, Ebersberg, Germany). The resulting sequences were analyzed using the SnapGenes tool (GSL Biotech LLC, San Diego, CA, USA). 

### 2.3. Construction of Lacticaseicin 30 Variant Plasmids for Expression in E. coli Cells

Each lacticaseicin variant plasmid was generated by site-directed mutagenesis using the pT7-6his-030 plasmid as template and the appropriate primers (Table S2), the QuikChange II Site-Directed Mutagenesis Kit (Agilent Technologies, Santa Clara, CA, USA), and following the recommended instructions.

### 2.4. Expression and Purification of Lacticaseicin 30 and Its Variants

Each plasmid constructed in this work was expressed in *E. coli* Rosetta and grown at 37 °C in LB broth, supplemented with ampicillin, until reaching the mid-log phase. Expression was then induced by adding 0.5 mM isopropyl β-D-1-thiogalactopyranoside (IPTG, Sigma Aldrich, St Louis, MO, USA), and the cells were incubated for three additional hours at 37 °C with shaking at 160 rpm. Cells were then harvested by centrifugation and resuspended in the Tris–HCl buffer (20 mM Tris–HCl pH 8, 300 mM NaCl). Finally, cells were lysed by sonication (OmniRuptor 4000 Ultrasonic Homogenizer, Omni International, Kennessaw, GA, USA) on ice-cold water and centrifuged at 11,000× *g* for 1 h. The supernatant was loaded onto a nickel resin grafted on a nitrilo-tri-acetic matrix (Protino Ni-NTA Agarose, Machery-Nagel, Düren, Germany) column previously equilibrated with Tris–HCl buffer. The nickel resin was washed with 2 × 10 column volumes of the same buffer supplemented with 30 mM imidazole and the bacteriocins were eluted using 5 column volumes of the previous buffer supplemented with 200 mM imidazole. A desalting step was performed using PD miditrap^TM^ columns (GE Healthcare Life Science, Pollard, UK) to remove imidazole. The histidine-tag was removed with Tev-protease (Sigma Aldrich, St Louis, MO, USA), while the TRX-tag of lacticaseicin 30 peptide variants (Nter-lacticaseicin 30, Cter-lacticaseicin 30 and Nter-H1-lacticaseicin 30) was removed with enterokinase (New England Biolabs, Ipswich, MA, USA). To separate the tag and the peptide without the tag, additional Ni-NTA chromatography was performed. The purity was verified by Tricine-SDS-PAGE [33]. When necessary, desalted bacteriocin suspensions were lyophilized using the freeze dryer Lyolab 3000 (Thermo Scientific, Waltham, MA, USA) following the recommended instructions. The final concentration of each purified peptide was determined with the bicinchoninic acid assay protein kit (BCA, Sigma Aldrich, St Louis, MO, USA), as recommended by the supplier.

### 2.5. Matrix-Assisted Laser Desorption Ionization Time-of-Flight Mass Spectrometry (MALDI-TOF MS)

Prior to MALDI-TOF-MS analysis, a purified Ser-lacticaseicin 30 suspension was concentrated using Pierce^TM^ C18 tips (Thermo Scientific, Waltham, MA, USA) according to the manufacturers’ instructions. MALDI-TOF mass-to-charge (*m*/*z*) ratios of Ser-lacticaseicin 30 were obtained using α-cyano-4-hydroxycinnamic acid (HCCA, 10 mg/mL) as matrix on an Autoflex Speed^TM^ (Bruker Daltonics, Bremen, Germany), running Flexcontrol 3.4 software (Bruker Daltonics). The Bruker bacterial test standard (Bruker Daltonics) was used to calibrate the mass spectrometer according to the manufacturer’s recommendations. Mass spectra were acquired in the positive linear ion mode across a *m*/*z* ratio of 2000–20000 Da using the manufacturer’s automatic method MBT_FC.par. The MALDI-TOF-MS spectra corresponded to an accumulation of 3000 laser shots, in 500 shot steps performed randomly on different areas of the spot. MALDI-TOF-MS spectra were managed using FlexAnalysis 3.4 software (Bruker Daltonics).

### 2.6. Reverse Phase-High Performance Liquid Chromatography (RP-HPLC) Coupled to Electrospray Ionization-Mass Spectrometry (ESI-MS)

Chloroform/methanol precipitation was performed, according to [34], from 200 µL of desalted Ser-lacticaseicin 30 suspension before RP-HPLC-ESI-MS analysis. Precipitated Ser-lacticaseicin 30 was redissolved in 40 µL of 25 mM ammonium bicarbonate buffer and 10 µL was chromatographically separated on an ACQUITY UPLC system (Waters Corporation) using a C18 column (150 × 3.0 mm, 2.6 µm, Uptisphere CS Evolution, Interchim, Montluçon, France). Eluent A was milliQ H_2_O containing formic acid (0.1%, *v*/*v*) and eluent B was acetonitrile (ACN) containing formic acid (0.1%, *v*/*v*). The ACN gradient (flow rate 0.5 mL/min) was as follows: from 5% to 40% eluent B over 45 min, from 40% to 95% eluent B over 5 min, followed by washing and equilibrating procedures with 95% and 5% solvent B for 5 min each, respectively. The eluate was directed into the electrospray ionization source of the Q-TOF Synapt G2-Si™ (Waters Corporation, Manchester, UK). MS analysis was performed in sensitivity, positive ion and data-dependent analysis (DDA) modes. The source temperature was set at 150 °C and the capillary and cone voltages were set to 3000 and 60 V, respectively. MS data were collected for *m*/*z* values in the range of 50 to 2000 Da with a scan time of 0.2 s and a lock mass correction with 556.632 *m*/*z*, corresponding to simply charged leucine encephalin. The mass spectrum corresponding to the sum of 400 ms acquisition was deconvoluted using UniDec software [35]. The molecular mass range was fixed to an upper limit of 20 kDa while the charge range was fixed between 1 and 50.

### 2.7. Analysis of the Ser-Lacticaseicin 30 Amino Acid Sequence by Peptide Fingerprinting

In total, 10 µL of native Ser-lacticaseicin 30, redissolved in 25 mM ammonium bicarbonate buffer, were hydrolyzed by 1 µL of trypsin/Lys-C mixture at 0.1 µg/µL for 3 h at 37 °C and again by adding another 1 µL of the enzyme mixture for 16 h at 37 °C and centrifuged for 10 min at 8000× *g*. The pellet was removed and the peptides in the supernatant were subjected to fingerprinting using the Q-TOF Synapt G2-Si™ spectrometer and the same HPLC and MS acquisition conditions as described above. A maximum of 10 precursor ions was chosen for MS/MS analysis with an intensity threshold of 10,000. MS/MS data were collected using the collision-induced dissociation (CID) mode and a scan time of 0.1 s at an energy collision of 8 V to 9 V for low *m*/*z* and at a range of 40 V to 90 V for high *m*/*z*.

For peptide identification, database searches were performed via PEAKS Studio Xpro software (Bioinformatics Solutions Inc., Waterloo, Canada) using the UniProtKB/Swiss-Prot-TrEMBL databases restricted to *Lactobacillus* (https://www.uniprot.org/, accessed on April 2022). Mass tolerance of 35 ppm and MS/MS tolerance of 0.2 Da were allowed. Data searches were performed, assigning trypsin as protease and three missed cleavage sites were allowed. Variable methionine oxidations were also considered. The relevance of peptide identities was judged according to their identification score returned by PEAKS Studio Xpro using a *p*-value of 0.05 (*p* < 0.05) and a false discovery rate (FDR) < 0.1%.

### 2.8. Alphafold2 Structure Prediction of Lacticaseicin 30 and Its Truncated Variants

AlphaFold2 [36] running locally at an Ubuntu 20.04 workstation and the full_dbs preset were used to predict structures based on the corresponding FASTA sequences of lacticaseicin 30, N-ter-lacticaseicin 30, N-ter-H1-lacticaseicin 30 and C-ter-lacticaseicin 30. AMBER99SB [37] relaxation and OpenMM [38] energy minimization were performed and the five models that were generated were ranked based on their pLDDT confidence score. The highest-ranked conformation was selected for each peptide and the structures were visualized using UCSF ChimeraX [39]. Structural alignments were also performed in ChimeraX using the maker command and the following default parameters: chain pairing:bb; Needleman–Wunsch alignment algorithm; BLOSUM-62 similarity matrix (Figure S1).

### 2.9. Circular Dichroism Spectroscopy

Circular dichroism measurements were performed in a 1 mm quartz cuvette using the JASCO 810 spectrophotometer (Jasco, Tokyo, Japan) without or with 10 mM of sodium dodecyl sulfate (SDS) micelle. Acquisition of the spectra was performed at 25 °C, in the wavelength range 190–260 nm for all samples and under nitrogen atmosphere. Before the measurement, the lyophilized bacteriocin was dissolved in phosphate buffer at pH 7 or pH 5 [40] to obtain a final concentration of 12 µg/mL. All spectra were recorded with appropriate blank subtractions and averaging of three independent measurements. The amounts of structural motifs were calculated using the BeStSel server [41]. Prediction of the secondary and three-dimensional structures of lacticaseicin 30 was performed using the I-Tasser tool [42,43].

### 2.10. Antimicrobial Activity

The antimicrobial activity of lacticaseicin 30 and its variants (500 µg/mL) was tested by the critical dilution micromethod using different target strains [44]. Samples were acidified to pH 5 and sterilized by filtration. Serial double dilutions of the filtrate samples were made in 200 µL volumes of BHI in a sterile 96-well Falcon microtiter plate (Corning, Tewksbury, MA, USA). Each well was inoculated with 2 µL of overnight culture of the target strain. Then, microplates were incubated at 37 °C for 24 h without agitation. Bacterial growth was estimated by measuring absorbance at 620 nm using a microtiter plate scanner (Biotech Instruments Inc., Winooski, VT, USA). The antibacterial activity was determined in arbitrary units per milliliter (AU/mL) according to the following formula: 2^n^ × (1000 µL/deposited volume), with *n* corresponding to the highest number of dilution at which growth inhibition of the sensitive strain is observed [45]. Therefore, the minimum inhibitory concentration (MIC) was directly determined from the bacteriocin activity and defined as the lowest concentration of bacteriocin resulting in no visible turbidity after 24 h of incubation.

## 3. Results

### 3.1. Heterologous Expression and Characterization of Lacticaseicin 30

**Figure 1:**
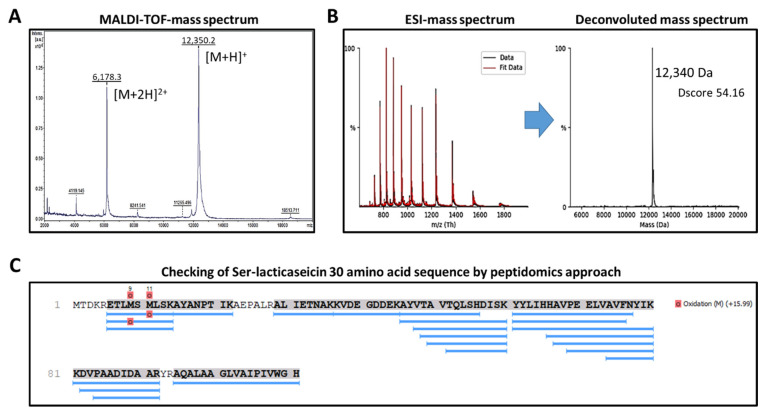
Mass spectrometry analyses of 6his-tag-depleted Ser-lacticaseicin 30 (average molecular mass 12,339.2 Da). (**A**) Linear positive MALDI-TOF mass spectrum illustrating the [M + H]^+^ and [M + 2H]^2+^ mass signals obtained. The measured MALDI-TOF-MS molecular mass was evaluated as 12,349.2 Da. (**B**) ESI mass spectrum and deconvoluted molecular mass (UniDec DScore: 54.16) revealing a molecular mass of 12,340 Da. (**C**) Peptide mapping of the 6his-tag-depleted Ser-lacticaseicin 30 after tryptic hydrolysis. Blue lines correspond to identified peptides along the lacticaseicin 30 sequence following the bioinformatic retreatment of RP-HPLC-MS/MS data using PEAKS Studio Xpro and the UniProtKB/Swiss-Prot-TrEMBL protein databases restricted to *Lactobacillus* (https://www.uniprot.org/, accessed on April 2022).

The pT7-6his-030 plasmid [30] was transferred to *E. coli* Rosetta strain. Production of lacticaseicin 30 was induced by addition of isopropylthio-β-galactoside (IPTG). Lacticaseicin 30 was purified by Ni-NTA chromatography, and the 6his-tag was removed using the TEV-protease. The purified and his-tag-depleted bacteriocin, produced by heterologous expression, was characterized by mass spectrometry (MS) (Figure 1) to confirm the bacteriocin amino acid sequence. According to the plasmid construction and the Tev-protease manufacturer’s instructions, the histidine-tag removal with Tev-protease leaves a serine residue at the N-terminal position (Ser-lacticaseicin 30), and therefore the calculated average theoretical molecular mass (MM) of the Ser-lacticaseicin is 12,339.2 Da (using ProtParam (https://web.expasy.org/protparam/, accessed on April 2022)). As shown in Figure 1A, the most intense mass signal (mass-to-charge ratio (*m*/*z*) of [M + H]^+^ bacteriocin ions = 12,350.2) and the second main mass signal (*m*/*z* = 6178.3) corresponding to [M + 2H]^2+^ bacteriocin ions matched well with the theoretical MM of the Ser-lacticaseicin 30. Consequently, the MALDI-TOF-MS measured MM was assessed as 12,349.2 Da for the heterologous expressed Ser-lacticaseicin 30. To accurately measure the molecular mass of Ser-lacticaseicin 30, this latter was concentrated by organic solvent precipitation, redissolved in ammonium bicarbonate buffer and subjected to reverse-phase high-performance liquid chromatography coupled to an electrospray ionization (ESI) high-resolution mass spectrometer. Figure 1B shows the ESI-MS spectrum and the corresponding deconvoluted mass spectrum, revealing an MM of 12,340 Da, confirming (i) that the purified and his-tag-depleted bacteriocin, produced by heterologous expression is the Ser-lacticaseicin 30, and (ii) that no further proteolytic maturation occurred. The entire amino acid sequence of the heterologously expressed lacticaseicin 30 was confirmed by peptide fingerprinting (Figure 1C). Indeed, after trypsin/Lys-C hydrolysis, the Ser-lacticaseicin-30-issued peptides were subjected to an RP-HPLC-MS/MS analysis, and the obtained LC-MS/MS data were submitted to PEAKS Studio Xpro for peptide identification against the UniProtKB/Swiss-Prot-TrEMBL databases restricted to *Lactobacillus* (accessed on April 2022). As shown in Figure 1C, PEAKS studio Xpro returned the identification of pre-bacteriocin from *Lactobacillus casei* with a protein identification score (−10logP) of 172.53, a sequence coverage of 88% for 20 identified peptides. Only three short-size amino acid sequences were not identified: (i) as expected due to the addition of a serine residue at the N-terminal extremity, the first five N-terminal amino acids; (ii) the six amino acid sequence AEPALR; and (iii) the dipeptide YR in the C-terminal region. Note that the methionine oxidations are due to the fingerprinting experimental procedure, and therefore the methionine residues of purified Ser-lacticaseicin are not oxidized. All together, these MS data evidenced that the amino acid sequence of the heterologously expressed, purified and His-tag-depleted bacteriocin corresponds to Ser-lacticaseicin 30.

The antimicrobial activity of the purified lacticaseicin 30 was tested at pH 5, with and without the 6×his-tag, and the MICs were determined using *E. coli* ATCC 8739 as the target strain. Lacticaseicin 30 displayed the same MIC value of 40 µg/mL, with and without the 6×his-tag.

### 3.2. Conformational Analysis of Lacticaseicin 30

**Figure 2:**
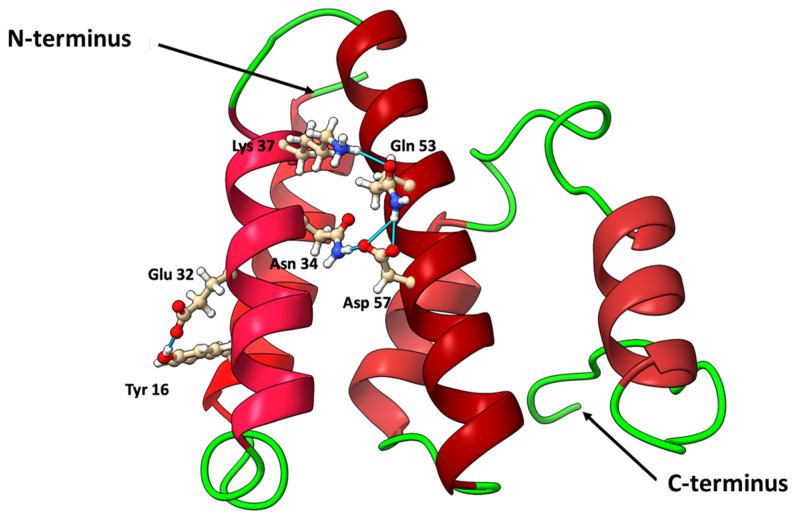
AlphaFold2 predicted structure of lacticaseicin 30 showing five helices (various shades of red) connected by coil regions (green). Five predicted H-bonds (blue) stabilize the position of the three helices.

**Figure 3:**
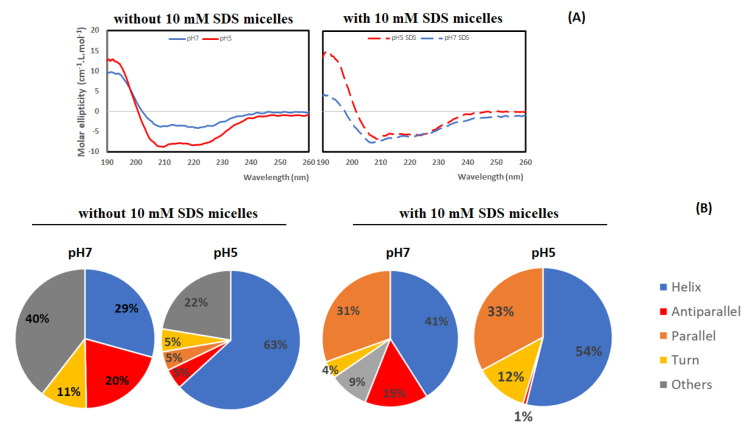
(**A**) Circular dichroism spectra of lacticaseicin 30 at pH 7 (blue) and pH 5 (red) recorded in the absence or presence of 10 mM SDS micelles. (**B**) The secondary structure content (%) of lacticaseicin 30 at pH 7 and 5 was predicted using the BestSel Software.

The secondary and three-dimensional (3D) structures of lacticaseicin 30 were predicted using AlphaFold2 [36], a machine learning approach that was proven to deliver highly accurate and reliable protein structure prediction [46]. As seen on Figure 2, the predicted secondary structure of lacticaseicin 30 is characterized by the presence of five helices (73 amino acids out of 111, i.e., 66%), several connecting coiled regions (38 amino acids out of 111, i.e., 34%) and five potential H-bonds indicating interactions between these distinct helical segments. The predicted largely helical structure was further confirmed by circular dichroism (CD). CD spectra were obtained at pH 7 and 5, in the presence or absence of sodium dodecyl sulfate (SDS) micelles used as a model of the anionic membrane bilayer [47]. The CD spectra showed that lacticaseicin 30 adopts a helical structure at both pH 5 and 7, and in the absence or presence of SDS micelles (Figure 3A). The content of the different secondary structures, in terms of helix, antiparallel and parallel β-sheets and turns, was evaluated using the BestSel software (Figure 3B). The percentage of α-helix in the presence of SDS micelles was 29% and 63% at pH 7 and 5, respectively, while it was estimated at 41% and 54%, respectively, in the absence of SDS micelles. These results show that interaction with a membrane-like environment is not required to trigger the organization of lacticaseicin 30 in a helical structure, which is intrinsically acquired even in buffer. Moreover, they show that an acidic pH environment favors a higher proportion of helix, suggesting it helps in folding the active structure of lacticaseicin 30 (Figure 3B).

### 3.3. Design of Lacticaseicin 30 Variants

**Figure 4:**
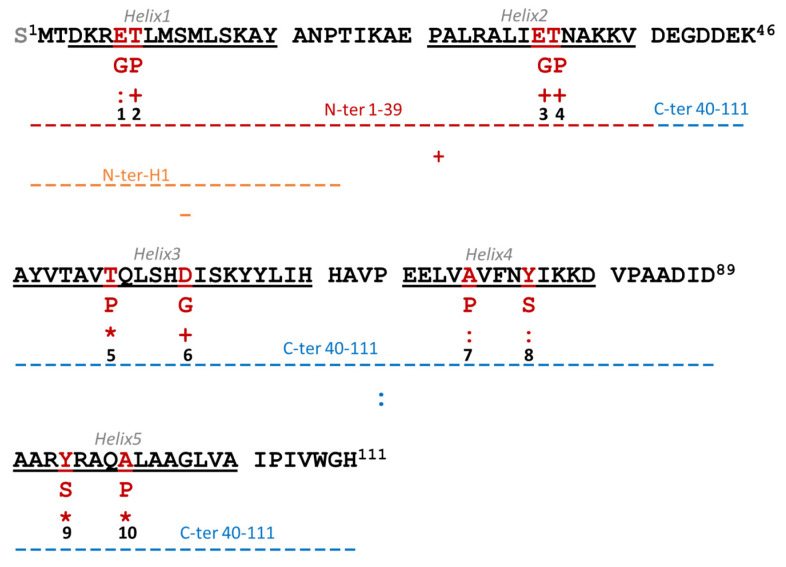
Amino acid sequence and predicted three-dimensional structure of lacticaseicin 30. The predicted helical regions (helix 1 to helix 5) are underlined. Truncated forms of lacticaseicin 30 are shown by colored dotted lines (red: N-ter-Lacticaseicin 30, orange: N-ter-H1-Lacticaseicin 30 and blue: C-ter-Lacticaseicin 30). Mutated amino acids of variants are in red. Lacticaseicin 30 variants generated by directed mutagenesis are designated by a number between 1 and 10 (1: E6G, 2: T7P, 3: E32G, 4: T33P, 5: T52P, 6: D57G, 7: A74P, 8: Y78S, 9: Y93S and 10: A97P). Activities in the same range as native lacticaseicin 30 and total absence of activity are shown by + and −, respectively; * and “:” indicate successively a gradual decrease of activity.

Lacticaseicin 30 variants were designed and constructed in order to locate the regions and amino acids specifically involved in the anti-Gram-negative activity, since such activity is rarely reported for LAB-bacteriocins. To this end, the DNA coding for the N-terminal region carrying the first two helices (from Met1 to Asp39), or the central and C-terminal regions carrying the last three helices (from Glu40 to His111) were cloned into the pET-32b plasmid, leading to the N-ter-lacticaseicin 30 and C-ter-lacticaseicin 30 variants, respectively (Figure 4). In addition, a shortened N-terminal region (from Met1 to Thr20) carrying only the first α-helix, designed as N-ter-H1-lacticaseicin 30 (Figure 4), was also expressed in the same system, considering nonetheless that thioredoxin (TRX) and 6his-tags located upstream of the multicloning site improve solubility of the variant peptides and their purification by the Ni-NTA chromatography.

In addition, several mutants were generated by site-directed mutagenesis, in order to identify the amino acids which, play a role in the anti-Gram-negative activity, and to understand the involvement of the predicted helical segments. To this purpose, charged (Glu, Asp) or aromatic (Tyr) amino acids located inside each α-helix were replaced by uncharged amino acids (Gly, Ser) (Figure 4 and Figure S1). Furthermore, amino acids located in the center of each helix were substituted with proline, which is known for its role in breaking or introducing kinks in helices due to the lack of amide proton (Figure 4 and Figure S1).

### 3.4. The N-Terminal Region Is Sufficient to Exert Anti-Gram-Negative Activity

After being produced, purified and quantified, lacticaseicin 30 and its variants, N-ter-lacticaseicin 30, C-ter-lacticaseicin 30 and N-ter-H1-lacticaseicin 30, were assessed for their antibacterial activity, which was determined at pH5 against four Gram-negative bacterial strains and *Listeria innocua* CIP 80.11 as Gram-positive bacterium (Table 1). Lacticaseicin 30 inhibited the growth of all target bacteria (Table 1), and particularly of *Escherichia coli* ATCC 8739 and *Proteus vulgaris* ATCC 33420, against which MIC values were the lowest (40 µg/mL), contrary to those obtained against *Pseudomonas aeruginosa* ATCC 27853 (160 µg/mL). The antibacterial activity against Gram-negative bacteria was clearly evidenced. Remarkably, the shortened variants, N-ter-lacticaseicin 30 and C-ter-lacticaseicin 30, both exhibited significant activity against the aforementioned Gram-negative target bacteria, as well as against the Gram-positive *Listeria innocua* CIP 80.11 (Table 1). The MIC values obtained with the N-ter-lacticaseicin 30 and C-ter-lacticaseicin 30 variants revealed that the N-terminal 1–39 region of lacticaseicin 30 was sufficient for the anti-Gram-negative activity against most of the targets (*P. aeruginosa* was no longer inhibited efficiently), whereas that of the truncated N-terminal peptide N-ter-H1-lacticasecin 30 was fully abolished, arguing that the first helix at the bacteriocin N-terminus is insufficient for the anti-Gram-negative activity.

### 3.5. Amino Acids Critical for the Anti-Gram-Negative Activity of Lacticaseicin 30

Different variants of lacticaseicin 30 were generated by site-directed mutagenesis using the pT7-6his-030 plasmid. These substitutions were performed in regions expected to play a key role in the folding of the bacteriocin. Importantly, these amino acid substitutions were introduced in the middle of each predicted α-helix in order to induce a conformational change. The antibacterial activity of lacticaseicin 30 and its variants was measured against the four selected Gram-negative bacteria (Table 2). Overall, amino acid substitutions led to a decrease in the antibacterial activity, except for E32G, T33P and D57G peptides; these MIC values remained unchanged and similar to those of lacticaseicin 30 (Table 2). Furthermore, when Glu6, Tyr78 and Tyr93 are mutated, they induce a net decrease of activity, suggesting a role in the anti-Gram-negative activity. Moreover, the MIC values obtained for lacticaseicin 30 and its truncated forms suggested that the Gram-negative activity (*E. coli*, *Salmonella*, *Proteus* and *Pseudomonas*) require the presence of helix 1. Helix 2 and its acidic residue E32 appeared to not be a determinant for the anti-Gram-negative activity evaluated in this study. Helix 3 and its aromatic residue T52 seems to be weakly required for the anti-Gram-negative activity. Helix 4 and its aromatic residue Y78 is, however, important for the anti-Gram-negative activity. Remarkably, helix 5 is important for activity against *Escherichia coli* and *Proteus* but is less important against *Salmonella* and *Pseudomonas* (Table 1 and Table 2).

## 4. Discussion

A notable number of AMPs including some bacteriocins exhibit potent and broad-spectrum antimicrobial activities. Some of them are cationic and perturb the permeability of the bacterial membrane bilayers. The presence of positive charges and hydrophobic residues constitutes a common trait of the AMPs which interact with the bacterial membrane, causing pores and membrane depolarization or altering the microbial metabolic pathways or inhibiting DNA/RNA/protein synthesis [40,48,49,50]. Bacteriocins can be bacteriostatic or bactericidal, induce a rapid killing effect and are thought to have a lower propensity to develop resistance than conventional antibiotics [10,20,49,51]. Overall, the modes of action of LAB-bacteriocins acting against Gram-positive bacteria have been well studied [8,28], but those used by LAB-bacteriocins acting against Gram-negative bacteria are much less documented [25,26,29], and remain to be fully explored.

Recently, we have isolated the *L. paracasei* CNCM I-5369 strain, and shown its potential to produce five distinct class II bacteriocins and inhibit Gram-negative bacteria, including strains of *E. coli* resistant to colistin [29]. Here, we focused on lacticaseicin 30, one of these bacteriocins, which was then used as a model to understand this original activity. Of note, its antibacterial activity may result from the combined action between the peptide itself and an acidic pH (pH 5) [29,30]. According to the classification proposed by Alvarez-Sieiro et al. [22], lacticaseicin 30 appeared to have the characteristics of class IIc bacteriocins. Indeed, this bacteriocin is synthesized without a leader sequence and does not undergo post-translational modifications, suggesting it could be a novel leaderless bacteriocin. Pérez-Ramos et al. [21] reported that leaderless bacteriocins disrupt the cell membrane of target bacteria and most of them do not require any docking molecule for their antimicrobial activity.

As with a few AMPs already reported [52,53], lacticaseicin 30 contains a higher number of negatively charged residues (Asp and Glu) than of positively charged ones (Arg and Lys), with an isoelectric point (p*I*) of 6.05, and many hydrophobic residues. At a neutral pH (pH > p*I*), the peptide is anionic while at an acidic pH (pH < p*I*), it is cationic. As previously mentioned, hydrophobic and cationic residues are one of the main characteristics of AMPs, and the presence of cationic residues can mediate interactions with negatively charged bacterial lipids, while the hydrophobic residues could contribute to the membrane perturbation [54].

The lacticaseicin 30 amino acid sequence is structurally organized into five distinct helices (H1 to H5) (Figure 2), based on the AlphaFold2 predictions and the circular dichroism spectroscopy data obtained in the absence or presence of SDS micelles at pH 5 or 7 (Figure 3).

As indicated in Table 1, the activity against Gram-negative bacteria appeared to be exerted in a strain-dependent manner. On the other hand, we observed an increase of the MIC values with the shortened peptide carrying the C-terminal region of lacticaseicin 30 (C-ter-lacticaseicin 30), in comparison with the native peptide (Table 1). Accordingly, the antibacterial susceptibility has decreased 4-fold against *E. coli* ATCC 8739 and 2-fold against *Salmonella enterica* serovar Newport ATCC 6962 and *Proteus vulgaris* ATCC 33420. Remarkably, these shortened analogs displayed similar activity against *Pseudomonas aeruginosa* ATCC 27853. It is worthy of note that antibacterial assays performed on different *E. coli* strains carrying or not carrying modifications on their LPS were conducted with novel bacteriocins including thereof the class II lacticaseicin 30 [29], and the results obtained did not suggest LPS as the main target of these novel class II bacteriocins. Nonetheless, the activity against *Listeria innocua* CIP 80.11, used as the Gram-positive target strain, remained unchanged. These results delineate the dual role of the N-terminal region composed of helix 1 and helix 2, while the C-terminal region and more particularly helices 4 and 5 could modulate the antibacterial activity according to the target bacteria, suggesting another mechanism of action that remains to be determined. This study is in line with that of Van Kraaij et al. [55], who showed the role of the C-terminal region of nisin across the membrane. Similarly, Johnsen et al. [56] and Rihakova et al. [57] revealed the involvement of the C-terminal region of pediocin-like bacteriocins (class IIa bacteriocins) in determining their antibacterial spectrum.

To gain further insights on the antibacterial activity of lacticaseicin 30, another analog named N-ter-H1-lacticaseicin 30, consisting of a shortened N-terminal region carrying the 1 to 20 amino acids and including only the first predicted α-helix, was designed, expressed and produced. Interestingly, the antibacterial activity remained unchanged against the Gram-positive target strain, whereas it was completely abolished against the Gram-negative target bacteria (Table 1). This result argues that at least two helices located in the N-terminal region are required for activity against Gram-negative bacteria. Furthermore, substitutions of selected amino acids have been performed by site-directed mutagenesis with the aim to understand their roles in the global antibacterial activity. Site-directed mutation refers to the redesign of natural antimicrobial peptides by adding, deleting or replacing one or several amino acid residues [58]. Therefore, two types of key mutations have been created. The first one consisted of replacing glutamic acid, aspartic acid or tyrosine by glycine or serine, and the second consisted of replacing threonine or alanine by proline. These mutations have been created inside each predicted α-helix. Then, the peptide variants E6G, T7P, D57G, A74P, Y78S, Y93S, A97P, E32G, T33P and T52P were tested for their activities against the target Gram-negative bacteria. Overall, they exhibited a loss in the antibacterial activity except for E32G, T33P and T52P, for which anti-Gram-negative activity remained unchanged (Table 2). Proline is a non-polar amino acid and proline-rich AMPs act differently from other AMPs. Indeed, some proline-rich AMPs have been shown to enter the bacterial cytoplasm through the inner membrane transporter SbmA instead of killing bacteria through membrane disruption [59]. Once in the cytoplasm, some proline-rich AMPs target ribosomes and block the binding of aminoacyl-tRNA to the peptidyltransferase center and interfere with protein synthesis [60], while others bind and inhibit DnaK [61]. In the case of lacticaseicin 30, which is devoid of proline residues, the substitution of T7, T52, A74 or A97 with prolines decreases the activity of the corresponding peptides, indicating that the mechanism implied in the anti-Gram-negative activity of lacticaseicin 30 is perturbed by the presence of proline. On the other hand, glycine is generally classified as a non-polar amino acid that induces flexibility of the peptide backbones [62,63].

## 5. Conclusions

To sum up, lacticaseicin 30 is predicted to adopt a secondary structure characterized by five helices. The generation of peptide variants carrying single point mutations, or truncated sequences, enabled some amino acids that play a major role in the structure of lacticaseicin 30 and its activity against Gram-negative bacteria, including strains of *E. coli* resistant to colistin. Moreover, this study permitted us to establish that at least two helices located in the N-terminal region are required for the anti-Gram-negative activity, while the C-terminal region would serve as a modulator of the activity, conferring its selectivity. These promising achievements open a new avenue in the characterization of LAB-bacteriocins endowed with activity against Gram-negative bacteria. Further experiments consisting of designing novel variants with enhanced antibacterial activity directed especially against resistant strains constitute our next goal.

## Figures and Tables

**Table 1:** Minimum inhibitory concentrations (MIC) values (µg/mL) of lacticaseicin 30 and its truncated forms.

Peptides	Molecular Mas (Da)	*Escherichia coli* ATCC 8739	*Salmonella enterica* Serotype Newport 6962	*Proteus* *vulgaris* ATCC 3342	*Pseudomonas aeruginosa* ATCC 27853	*Listeria* *innocua* CIP 80.11
Lacticaseicin 30	12,252.09	40	80	40	160	100
N-ter Lacticaseicin 30	4236.03	40	80	40	320	100
C-ter Lacticaseicin 30	8034.08	160	160	160	320	100
N-ter-H1 Lacticaseicin 30	1905.27	-	-	-	-	100

**Table 2:** Minimum inhibitory concentrations (µg/mL) of lacticaseicin 30 and its variant peptides.

Peptides	Molecular Mass (Da)	Putative Helix	*Escherichia coli* ATCC 8739	*Salmonella* Newport ATCC 6962	*Proteus* *vulgaris* ATCC 33420	*Pseudomonas aeruginosa* ATCC 27853
Lacticaseicin 30	12,252.09	-	40	80	40	100
E6G	12,180.03	H1	100	160	100	400
T7P	12,248.10	H1	60	160	60	200
E32G	12,180.03	H2	40	80	40	100
T33P	12,248.10	H2	40	80	40	100
T52P	12,248.10	H3	70	140	70	200
D57G	12,194.06	H3	40	80	40	100
A74P	12,278.13	H4	100	160	100	200
Y78S	12,176.00	H4	100	200	100	400
Y93S	12,176.00	H5	70	140	70	200
A97P	12,278.13	H5	60	100	60	200

## Data Availability

All data generated or analyzed during this study are included in this published article.

## Supplementary Materials

The following supporting information can be downloaded at: https://www.mdpi.com/article/10.3390/pharmaceutics14091921/s1, Table S1: Bacterial strains and plasmids used in this study; Table S2: Sequences of oligonucleotide primers used in this study; Figure S1: Structural alignment of the predicted structures of native lacticaseicin 30 (brown) and its truncated derivatives N-ter-lacticaseicin 30 (transparent red, RMSD between 37 pruned atom pairs 0.381 angstroms; across all 39 pairs: 1.535 angstroms), N-ter-H1- lacticaseicin 30 (transparent green, RMSD between 20 pruned atom pairs 0.797 angstroms; across all 20 pairs: 0.797 angstroms) and C-ter-lacticaseicin 30 (transparent blue, RMSD between 31 pruned atom pairs 0.888 angstroms; across all 73 pairs 8.364 angstroms). Substituted amino acids in the variant peptides are visible as balls and sticks in their position in the native peptide.
