# Supplementary material for: Structure of Lacticaseicin 30 and Its Engineered Variants Revealed an Interplay between the N-Terminal and C-Terminal Regions in the Activity against Gram-Negative Bacteria
Source: Pharmaceutics. 2022 Sep 12;14(9):1921. doi: 10.3390/pharmaceutics14091921 (PMC9505257; doi:10.3390/pharmaceutics14091921)
Supplement: Supplementary file 1 [file pharmaceutics-14-01921-s001.zip › pharmaceutics-1823575-supplementary.pdf]

Supplementary Materials

Table S1. Bacterial strains and plasmids used in this study.

Strains and Plasmids	Relevant characteristics	References
**Strains**		
	**Strains used for heterologous expression and cloning**	
Escherichia coli JM109	*recA1. endAl, gyrA96, thi. hsdR17, supE44, relA1, l-, A(iac-proAB), [F', traD36, proAB, iacI^{q}ZAM15]*	Promega, Madison, United States
Escherichia coli Rosetta	*[F´, traD36, ΔompP, proA$^+$B$^+$, lacIq, Δ(lacZ)M15]ΔompT, endA1, recA1, gyrA96 (Nal^r), thi-1, hsdR17 (r_K^-, m_K^+), e14$^-$ (McrA$^-$), relA1, supE44, Δ(lac-proAB), Δ(rhaBAD)::T7 RNA polymerase*	Promega, Madison, United States
	**Strains used as targets for antimicrobial activity tests**	
Escherichia coli ATCC 8739		[29](5)
Listeria innocua CIP 80.11		[64](62)
Proteus vulgaris ATCC 33420		(67)
Salmonella enterica Serotype Newport ATCC 6962		(52)
Pseudomonas aeruginosa ATCC 27853		(19)
	**Plasmids**	
pT7-6his-030	*pT7, His-tag, Tev-site, orf030, Amp^R, LacI, LacO*	(5)
pET-32b(+)	pT7, His tag, Trx-tag, S-tag, Amp^R, LacI, LacO, MCS	MercK Millipore, Burlington, United States
pET-32b-030	pET-32b(+) derivative carrying a 333 pb DNA fragment from pT7-6his-030 with *orf030* gene	In this study
pET32b-Nter_030	pET-32b(+) derivative carrying a 117 pb DNA fragment from pT7-6his-030 with *orf030* gene fragment	In this study
pET32b-Cter_030	pET-32b(+) derivative carrying a 216 pb DNA fragment from pT7-6his-030 with *orf030* gene fragment	In this study
pET32b-Nter-H1_030	pET-32b(+) derivative carrying a 81 pb DNA fragment from pT7-6his-030 with *orf030* gene fragment	In this study

pT7: T7 promoter, *Amp^R*: ampicillin resistant, *LacI* : lacI coding sequence, *LacO* : lac Operator

Table S2. Sequences of oligonucleotide primers used in this study.

Name	Sequence 5' → 3'	Use
**F-*BamHI*-030**	AAAA**GGATCC**ATGACAGACAAACGTGAAACTT	Amplification of *orf030* up-stream fragment
**R-030-*HindIII***	AAAA**AAGCTT**CTAATGCCCCCAAACAATGG	Amplification of *orf030* down-stream fragment
**R-Nter_030-*HindIII***	AAAA**AAGCTT**CTAATCAACCTTTTTGGCGTTGGT	Amplification of *orf030_nter* down-stream fragment
**F-*BamHI*-Cter_030**	AAAA**GGATCC**ATGGAAGGTGATGACGAAAAGGC	Amplification of *orf030_cter* up-stream fragment
**R-Nter-H1_030-*HindIII***	AAAA**AAGCTT**TTACGTTGGATTAGCGTATGCC	Amplification of *orf030_nter-h1* down-stream fragment
**F-030_mutE6G**	ACAGACAAACGTGGAACTTTAAT	to make mutations in the pT7-6his-030 plasmid in
**R-030_mutE6G**	ATTAAAGTTCCACGTTTGTCTGT	order to replace the Glu 6 to Gly in lacticaseicin 30
**F-030_mutT7P**	AAACGTGAACCTTTAATGTCG	to make mutations in the pT7-6his-030 plasmid in
**R-030_mutT7P**	CGACATTAAAGGTTCACGTTT	order to replace the Thr 7 to Pro in lacticaseicin 30
**F-030_mutE32G**	GCGCTTATCGGAACCAACG	to make mutations in the pT7-6his-030 plasmid in
**R-030_mutE32G**	CGTTGGTTCCGATAAGCGC	order to replace the Glu 32 to Gly in lacticaseicin 30
**F-030_mutT33P**	GCTTATCGAACCCAACGC	to make mutations in the pT7-6his-030 plasmid in
**R-030_mutT33P**	GCGTTGGGTTCGATAAGC	order to replace the Thr 33 to Pro in lacticaseicin 30
**F-030_mutT52P**	CGGCTGTCCCTCAGTT	to make mutations in the pT7-6his-030 plasmid in
**R-030_mutT52P**	AACTGAGGGACAGCCG	order to replace the Thr 52 to Pro in lacticaseicin 30
**F-030_mutD57G**	GTTGTCCCACGGCATTTCC	to make mutations in the pT7-6his-030 plasmid in
**R-030_mutD57G**	GGAAATGCCGTGGGACAAC	order to replace the Asp 57 to Gly in lacticaseicin 30
**F-030_mutA77P**	CTGGTGCCCGTCTTCA	to make mutations in the pT7-6his-030 plasmid in
**R-030_mutA77P**	TGAAGACGGGCACCAG	order to replace the Ala 77 to Pro in lacticaseicin 30
**F-030_mutY78S**	CCGTCTTCAATTCCATCAAAAA	to make mutations in the pT7-6his-030 plasmid in
**R-030_mutY78S**	TTTTTGATGGAATTGAAGACGG	order to replace the Tyr 78 to Ser in lacticaseicin 30
**F-030_mutY93S**	GCACGTTCTCGCGCGCAA	to make mutations in the pT7-6his-030 plasmid in
**R-030_mutY93S**	TTGCGCGCGAGAACGTGC	order to replace the Tyr 93 to Ser in lacticaseicin 30
**F-030_mutA97P**	GCGCAACCCTTGGC	to make mutations in the pT7-6his-030 plasmid in
**R-030_mutA97P**	GCCAAGGGTTGCGC	order to replace the Ala 97 to Pro in lacticaseicin 30
**T7**	TAATACGACTCACTATAGGGGA	Cloning verification in pET-32(+) plasmid
**T7 term**	GCTAGTTATTGCTCAGCGG	

Supplementary Figure

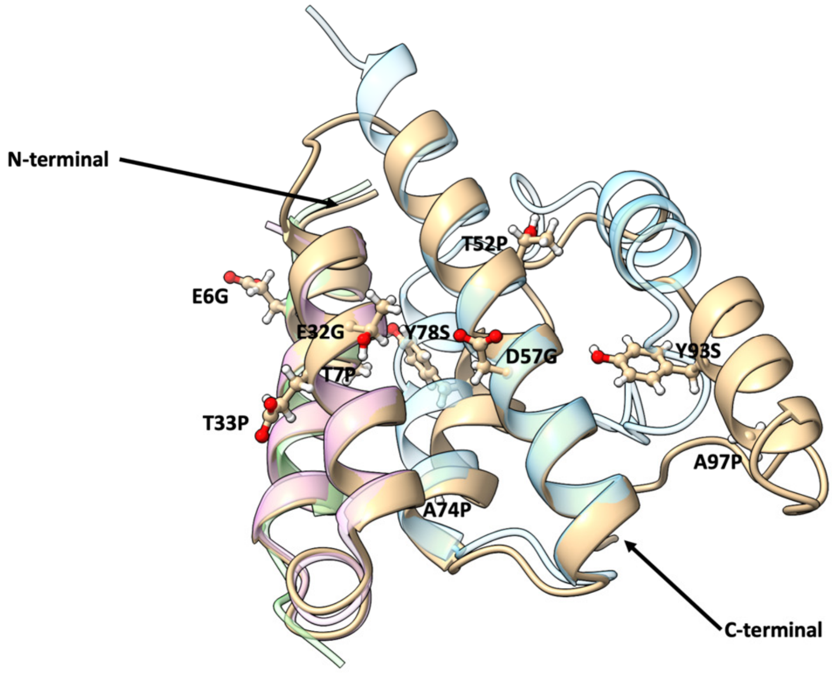

Figure S1. Structural alignment of the predicted structures of native lacticaseicin 30 (brown) and its truncated derivatives N-ter-lacticaseicin 30 (transparent red, RMSD between 37 pruned atom pairs 0.381 angstroms; across all 39 pairs: 1.535 angstroms), N-ter-H1- lacticaseicin 30 (transparent green, RMSD between 20 pruned atom pairs 0.797 angstroms; across all 20 pairs: 0.797 angstroms) and C-ter-lacticaseicin 30 (transparent blue, RMSD between 31 pruned atom pairs 0.888 angstroms; across all 73 pairs 8.364 angstroms). Substituted amino acids in the variant peptides are visible as balls and sticks in their position in the native peptide.
